# Mpox in East Africa: Learning from COVID-19 and Ebola to Strengthen Public Health Responses

**DOI:** 10.3390/v16101578

**Published:** 2024-10-08

**Authors:** Pierre Gashema, Tumusime Musafiri, Felix Ndahimana, Hyppolyte Iradukunda, Eric Saramba, Stuart T. Nyakatswau, Noel Gahamanyi, Patrick Gad Iradukunda, Ayman Ahmed, Tafadzwa Dzinamarira, Claude Mambo Muvunyi

**Affiliations:** 1 Department of Research, Repolicy Research Centre, Kigali P.O. Box 7584, Rwanda; 2 Centre for Genomic Biology, INES Ruhengeri, Ruhengeri P.O. Box 155, Rwanda; 3 Partners in Health, Kigali P.O. Box 3432, Rwanda; 4 Laboratory of Entomology, Wageningen University and Research, Droevendaalsesteeg 4, 6708 PB Wageningen, The Netherlands; 5 College of Medicine and Health Sciences, University of Rwanda, Kigali P.O. Box 4285, Rwanda; 6 Research and Development Department, Wastinnova Pvt Ltd., 5/39 Strachan St, Harare, Zimbabwe; 7 Rwanda Biomedical Centre, Kigali P.O. Box 7162, Rwanda; 8 Drugs Department, Rwanda Food and Drugs Authority, Kigali P.O. Box 1948, Rwanda; 9 Faculty of Science, University of Basel, Petersplatz 1, Basel P.O. Box 4001, Switzerland; 10 Institute of Endemic Diseases, University of Khartoum, Khartoum 11111, Sudan; 11 ICAP Columbia University, Lusaka, Zambia; 12 School of Health Systems and Public Health, University of Pretoria, Pretoria 0002, South Africa

**Keywords:** epidemic, pandemic, poxvirus, Ebola virus, COVID-19, global health security, mpox virus, mpox vaccine

## Abstract

The Africa Centers for Disease Control and Prevention declared mpox a Public Health Emergency of Continental Security (PHECS) in Africa. African public health systems have moved to mobilize a response against a backdrop of inherent significant challenges. With this commentary, we discuss how lessons from past public health emergencies, particularly COVID-19 and Ebola outbreaks, have prepared the region for improved disease surveillance, rapid response strategies, and effective public health communication and how these lessons can be applied to the mpox response, emphasizing the importance of strong healthcare infrastructure, effective data sharing, community engagement, targeted interventions, and robust contact tracing. Additionally, addressing misinformation and building public trust are crucial for controlling the spread of any disease. By leveraging these strategies, African countries can enhance their response to mpox. This includes improving diagnostic capabilities, strengthening cross-border collaborations, and prioritizing vaccination campaigns where needed. Ultimately, by applying the hard-earned lessons from the COVID-19 pandemic and Ebola outbreak, the East Africa region can better address the challenges posed by mpox and safeguard public health.

## 1. Introduction

Mpox (formerly known as monkeypox) is a viral zoonotic disease that is caused by Mpox virus (MPXV). MPXV is a member of the *Poxviridae* family and belongs to the *Orthopoxvirus* genus, and it was first identified in 1958, with the first human case of mpox reported in the Democratic Republic of Congo (DRC) a decade later in 1970 [1]. In Africa, the local transmission of mpox has been documented in several countries, including the DRC, Gabon, Cameroon, Ivory Coast, Sierra Leone, Liberia, Benin, the Central African Republic, Congo, and South Sudan [2]. The circulating virus is classified into three distinct clades: Clade I, found in the Congo Basin, is associated with up to 10% mortality in humans and is primarily transmitted by rodents with minimal human-to-human transmission. Clade IIa, endemic to West Africa, exhibits low mortality and is also zoonotic in nature. Clade IIb, however, is currently spreading worldwide, primarily through human-to-human transmission [3]. The existence of a divergent lineage within Clade I has been observed in the eastern part of DRC, Rwanda, Burundi and Uganda [4,5].

**Figure 1:**
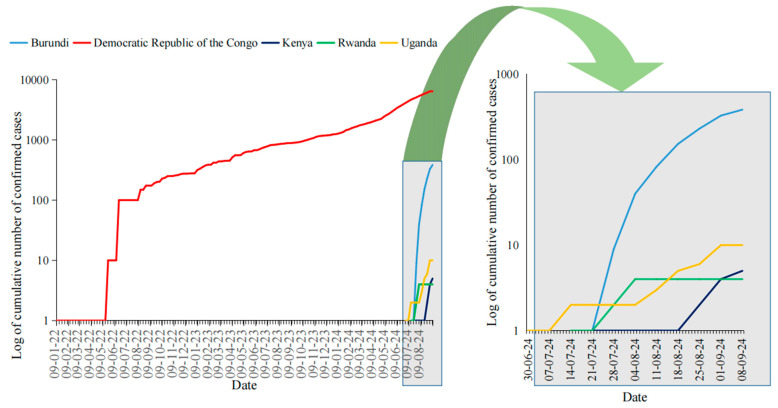
Mpox confirmed cases in DRC, Burundi, Rwanda, Uganda and Kenya. This figure demonstrate the cumulative number of the confirmed mpox virus cases in East Africa expressed in logarithm of base 10. Due to the logarithmic expression of the data, the period in which the surveillance was already established through testing without the identification of cases was included, but the data are not plotted as 0 cannot be expressed. The cases in the DRC have been increasing significantly, followed by Burundi, while other countries like Rwanda, Kenya, and Uganda experience fewer cases.

Since 2022, mpox cases have been steadily increasing in the DRC, and they raised sharply a year later and spread in the region, with the neighboring countries including Burundi, Uganda, Rwanda, and Kenya declared mpox outbreaks in July 2024. Figure 1 below shows the reported cases in the East African region. By then, over 37,583 cases and 1451 deaths had been reported in African Union member states. On 13 August 2024, the Africa Centers for Disease Control and Prevention (Africa CDC) declared mpox a public health emergency of continental security [6].

Based on the genomic similarity and cross-immune protection, it is assumed that the emergence of mpox in Africa is linked to the decline in smallpox vaccination following its eradication in 1980 [7]. Additional major contributing factors include environmental and socioeconomic factors: a wide range of susceptible hosts, changes in climate, living environment, and land use and cover, increased population size and dynamics, increased cross-species contacts, and globalization were theorized as major drivers of mpox transmission [8]. These factors create a complex landscape that makes mpox more prevalent in Africa [7,8].

Mpox is a DNA viral disease transmitted primarily through physical contact with an infected individual’s skin, saliva, or mucous membranes. This zoonotic disease can also spread through direct contact with infected hosts including humans, domestic animals, wild animals, the consumption of raw infected animal products, and contaminated materials [9]. Particularly men who have sex with men are thought to be the main driver of this viral transmission; crowded living conditions and sharing utensils also increase the risk of transmission [10,11]. The ongoing outbreak in the DRC associated with Clade Ib has been linked to sex workers in a mining town in North Kivu. This underscores the role of sex workers in the transmission of mpox [12].

The prevention of mpox mainly relies on vaccination, community engagement, and behavior change [13,14]. Although little information is available about the effectiveness of the currently available vaccines against the newly emerging clade of MPXV, considering the increasing mortality rate associated with this variant, vaccines should be experimentally administered to individuals at high risk, as they can reduce the course of the outbreak [15]. Nevertheless, this should be accompanied by close monitoring and evaluation with supportive care in place to intervene whenever needed. On the other hand, more investment should be made in preventive measures mainly including community engagement and health education to raise awareness, empowering communities to claim ownership and leadership in surveillance, prevention, and control. Particularly, improving sanitation and hygiene, practicing safe sex, and avoiding contact with and the consumption of infected animal products were proven cost-effective and sustainable prevention and control measures. The implementation of community-led syndromic surveillance can play a crucial role, as an early warning system that triggers the response from stakeholders about risk factors, transmission, and preventive measures of mpox is equally crucial. Avoiding close contact with wildlife as well as frequent hand cleaning with soap and water or an alcohol-based hand rub is advised [9]. The long-term containment of the mpox outbreak in Africa requires a coordinated effort across all levels of public health response. It is critical for African countries to address gaps in diagnostics, transmission control, and vaccine development by investing in research, healthcare infrastructure, and human resources [11].

Mpox presents unique diagnostic challenges with polymerase chain reaction (PCR) being the only validated and recommended testing option [16]. The main challenges persist in the sensitivity and specificity of easy-to-use tests, such as antigen and antibody detection methods, which are primarily utilized as auxiliary tools in mpox diagnosis. However, these conventional techniques detect orthopoxviruses (OPXVs) but exhibit low sensitivity due to significant serological cross-reactivity [17]. On the other hand, serum antibody testing can evaluate vaccine efficacy or support epidemiological studies; however, due to the cross-reactivity of OPXV antibodies, serological tests lack specificity for mpox virus (MPXV) infection [17]. More importantly, due to the virus evolution and growing rate of mutations in the genomic region targeted by the recommended and commonly available PCR kits, the molecular detection itself is not fully reliable, and it can give false negative results [18].

While PCR is accurate, it requires well-trained personnel and advanced infrastructure, limiting its availability to national levels [18,19]. As observed during the COVID-19 pandemic, the inability to test at peripheral levels and health centers hinders timely case detection, treatment and follow-up, underscoring the need for easy-to-use rapid tests [20].

The recent mpox outbreak in East Africa has underscored the ongoing challenges faced by health systems in Africa regarding the preparedness, prevention, and response to emerging infectious diseases. Despite the region’s experience with previous outbreaks like COVID-19 and Ebola, mpox presents a unique set of challenges that require tailored strategies. In this commentary, we discuss the potential tools available within the East Africa region to respond to mpox, leveraging the existing infrastructure used for COVID-19 and Ebola response efforts.

## 2. Most at Risk Population Groups

According to the WHO, healthcare providers, people involved in burial ceremonies, persons in direct contact with infected animals including those deal with games meat, individuals in close contact such as sexual or caring for Ebola patient, and infant of infected and recovering pregnant women are at particularly high risk of Ebola [21] While, for COVID-19, elder population and people with pre-existed medical conditions were at higher risk of developing severe disease. On the other hand, pregnant and breastfeeding women and their infants, people living with HIV, and males who have sex with males are at higher risk of MPXV [22]. Pregnant women, in addition to the risk of vertical transmission of the virus to their fetus through placenta or infant through breastfeeding, they are also at risk of stillbirth and miscarriage [23].

## 3. Brief Overview of Testing Capacity in East Africa Community (EAC) Region: Implication for Mpox Response

The EAC has collaboratively made significant strides in strengthening its diagnostic capacity, particularly in response to recent outbreaks like Ebola, COVID-19, and Rift Valley fever. This enhanced capacity has proven invaluable in the response to the ongoing mpox outbreak. In Rwanda, the Biomedical Center (RBC) has invested in advanced diagnostic capabilities, including incorporating the genomics analysis into the collaborative surveillance. This has enabled the early detection and characterization of circulating mpox strains, specifically Clade Ib MPXV. Similar to Rwanda, Burundi has developed domestic testing capacity and is utilizing genomic analysis to track mpox strains [23,24]. The DRC has expanded its testing infrastructure nationwide, particularly in response to Ebola and COVID-19 [25,26]. The National Institute of Biomedical Research (INRB) has been upgraded, and technicians have received training to handle various infectious diseases [27]. Similarly, Uganda has significantly improved its testing capacity, leveraging mobile laboratories and advanced infrastructure [28]. The country’s community-based diseases surveillance and contact tracing model strategies have further strengthened its response capabilities [29]. Kenya has benefited from existing infrastructure, including the Kenya Medical Research Institute (KEMRI), and it has successfully utilized the EAC mobile laboratory [30]. The EAC mobile laboratories have played a pivotal role in the region’s outbreak response. These biosecurity level 3/4 laboratories offer rapid diagnostic capabilities, reducing turnaround times, and enabling timely interventions [31]. Their portability and flexibility have been crucial in reaching remote areas and hotspots, ensuring the prompt detection and containment of infectious diseases, including mpox.

The enhanced testing capacity in EAC countries, coupled with the capabilities of the EAC mobile laboratories, has positioned the region well to respond to the mpox outbreak. Key implications include early detection and containment, regional collaboration, and enhanced preparedness. Rapid testing allows for the early identification of cases, enabling swift isolation and contact tracing to prevent further transmission [31]. The EAC Mobile Laboratories have fostered regional cooperation, facilitating standardized testing protocols and data sharing. The experience gained from responding to previous outbreaks, such as Ebola and COVID-19, has strengthened the region’s preparedness for future health emergencies. The EAC’s investment in testing capacity and the deployment of mobile laboratories have been instrumental in mitigating the impact of infectious diseases. These efforts are critical for effectively managing the ongoing mpox outbreak and ensuring the region’s public health resilience.

Countries are actively implementing a range of interventions to address the mpox outbreak. Drawing on lessons learned from the COVID-19 pandemic can significantly enhance the effectiveness of these measures and help prevent the further spread of mpox across the continent [32]. Robust coordination among global, regional, national, and local health agencies, coupled with strong political backing, is essential for effective mpox response [32,33,34]. Securing sustainable funding for prevention initiatives and providing comprehensive training for healthcare providers, epidemiologists, and community health workers are critical components of an effective strategy. Rwanda’s 4 × 4 reform, which aims to quadruple its health workforce development, offers a valuable lesson for other EAC member states and other countries with similar settings to adopt [35]. Additionally, efforts to educate and prepare communities are crucial in ensuring readiness and effective response to mpox [36].

Vaccines are essential for controlling the mpox outbreak as it is recommended by African CDC and WHO for prevention and response to the disease outbreaks [37]. Studies have shown that the smallpox vaccine is effective against mpox with a reported efficacy of 78% following a single dose [38]. While some African countries, such as South Africa and Egypt, have demonstrated their ability to produce vaccines, as observed during the COVID-19 pandemic, vaccine inequality remains a significant challenge in the region [25]. A 2022 Africa CDC report indicated that around 1.2 billion people in Africa had no access to the mpox vaccine [39].This disparity in vaccine availability and accessibility hinders efforts to control the mpox outbreak on the continent [40,41]. Since 2024, the DRC has become the epicenter of mpox with 4901 cases and 629 deaths reported [42]. In response, the Africa CDC, with support from UNICEF, delivered over 200,000 doses of the JYNNEOS mpox vaccine to the DRC to protect people at risk on the 5th and 7th September 2024 [42]. The same rapid intervention has been a cornerstone in responding to COVID-19 in African countries at risk, emphasizing the need for equal access to vaccines for those in need with the goal of containing pandemics.

## 4. Future Considerations

### 4.1. Strengthening Surveillance Systems

The massive biodiversity in Africa’s including pathogens populations such as viruses, parasites, bacteria, and fungi that infect both humans and animals as well as susceptible hosts underscores the urgent need for a robust surveillance system [43]. Strong surveillance is essential for effectively responding to outbreaks; integrating surveillance systems is crucial, since diseases do not recognize borders and must be closely monitored to maintain control efforts [44]. Additionally, expanding cross-border collaboration will facilitate data sharing and access to human resources and expertise, which have been lacking in our region [45].

### 4.2. Capacity Building and Healthcare Infrastructure

Healthcare providers have been at the forefront of the response to previous outbreaks, such as Ebola and COVID-19 [46]. Africa should rely on the long-term investment in the health development workforce as potential tools for future pandemic management [47]. Protecting them from contacting the disease and the availability of essential medical supplies will be the driving force for the success of future pandemics [48]. Strengthening supply chains to ensure timely access to personal protective equipment, vaccines, and medicines is vital for Africa’s preparedness [49]. More importantly, speeding up local pharmaceutical manufacturing and promoting research and development is believed to have an impact in addressing the gaps in equitable access to essential medicines and vaccines during outbreaks and pandemics [50,51].

### 4.3. Community Engagement and Education

Health literacy is essential for equipping communities with the knowledge needed to adhere to public health measures [52]. Given the cultural context in Africa, community-wide understanding of the importance of compliance is crucial. The success of community mobilization during COVID-19 vaccination campaigns demonstrates the potential impact of such efforts [53]. Future initiatives should focus on addressing vaccine hesitancy and other public health concerns through community meetings and awareness campaigns.

### 4.4. Mpox in Pregnant Women, Fetuses, and Newborns

Mpox can infect both the placenta and fetus, leading to congenital infections. During the 2023–2024 outbreak in the South Kivu Province, eight pregnant women were diagnosed with Clade I MPXV with 50% experiencing miscarriages or stillbirths [23]. Previous reports have also shown Clade I MPXV’s ability to cause significant fetal loss [23]. Historical data link smallpox, a related virus, to a 39.9% rate of miscarriage or premature birth, suggesting similar risks for mpox [54] These findings highlight the urgent need to prioritize pregnant women in high-risk groups for mpox vaccination to protect both mothers and infants.

### 4.5. Enhancing One Health Coordination

Considering the zoonotic nature of MPXV, investigating the host range locally in each country is essential for successful containment. The diversity and distribution of animal species susceptible to MPXV infections vary across Africa, and some alternative hosts may play a significant role in disease transmission. Wild animals were associated with the introduction and development of the first mpox outbreak outside Africa in 2003, highlighting the importance of considering animal reservoirs. In response to this and the overall increase in the emergence and spread of zoonoses such as Rift Valley fever, the Rwanda Biomedical Center is championing the institutionalization of a multisectoral transdisciplinary One Health strategy to strengthen the Global Health Security in the country, region, and internationally [55,56].

### 4.6. Establishing a Regional Hub for Pandemic Preparedness in Africa

The currently persistent challenge in availability and access to adequate diagnostic, prevention, and control tools including Persona Protection Equipment (PPE), lab kits and consumables, medication, vaccinations, and other logistics and medical supplies in Africa are urging the need for capitalizing on domestic investments and capacity building. Keep in mind that Africa is rich with technical expertise evident by the documented success in managing health emergencies and eradicating several neglected tropical diseases. However, because of the little investment in infrastructure, working environment, and limited opportunities for career growth, the region is suffering from severe brain drain [57]. This highlights the crucial need for a establishing regional hub for pandemic preparedness, prevention, and response that include a reference center of excellence for capacity building in the research, preparedness, prevention, and response to hemorrhagic fevers and high biocontainment pathogens in the region. Also, it should contain a work of art clinical facility for isolation, high-quality healthcare and case management, and clinical research. Enhancing the local manufacturing of diagnostic tools, lab consumables, therapeutic drugs and vaccines should be the top priority in the structure of such a hub. The establishment of such a regional hub might require engaging the leading stakeholders in Africa including the Africa CDC, Intergovernmental Authority on Development (IGAD), EAC, and the World Bank.

## 5. Conclusions

Lessons from past public health emergencies like COVID-19 and Ebola in East Africa have provided the region with valuable experience in the implementation of robust surveillance, effective response, and community engagement through public health communication. Advancements in these core pillars of pandemic preparedness, prevention, and response have significantly improved the region’s ability to manage the ongoing mpox outbreak if adequate resources were secured. Such resources should be prioritized to strengthening health systems including reinforcing healthcare infrastructure, enhancing diagnostic capabilities, and improving vaccination availability. Addressing misinformation and fostering public trust remain crucial components for successful outbreak control, ensuring that prevention and treatment strategies reach the most vulnerable populations. Strengthening the implementation of multisectoral and transdisciplinary One Health, cross-border coordination and collaboration will boost the success of preparedness, prevention, and response measures in the region. However, this could not be achieved without impactful private–public partnerships, community ownership, and the proper governance of public and global health in the region.

## Data Availability

The data used in this work are available upon request from the corresponding author.

## References

[1] Mpox (Formerly Monkeypox): Pathogenesis, Prevention and Treatment|Signal Transduction and Targeted Therapy. https://www.nature.com/articles/s41392-023-01675-2.

[2] Multi-Country Monkeypox Outbreak in Non-Endemic Countries. https://www.who.int/emergencies/disease-outbreak-news/item/2022-DON385.

[3] Virulence Differences of Mpox (Monkeypox) virus Clades I, IIa, and IIb.1 in a Small Animal Model—PubMed. https://pubmed.ncbi.nlm.nih.gov/36787354/.

[4] Clade I Mpox in Central and Eastern Africa—Level 2—Practice Enhanced Precautions—Travel Health Notices|Travelers’ Health|CDC. https://wwwnc.cdc.gov/travel/notices/level2/clade-1-mpox-central-eastern-africa.

[5] Sustained Human Outbreak of a New MPXV Clade I lineage in Eastern Democratic Republic of the Congo|Nature Medicine. https://www.nature.com/articles/s41591-024-03130-3.

[6] Mpox—African Region. https://www.who.int/emergencies/disease-outbreak-news/item/2024-DON528.

[7] Van Dijck C., Hoff N.A., Mbala-Kingebeni P., Low N., Cevik M., Rimoin A.W., Kindrachuk J., Liesenborghs L. (2023). Emergence of mpox in the post-smallpox era—A narrative review on mpox epidemiology. Clin. Microbiol. Infect..

[8] Ogunleye S.C., Akinsulie O.C., Aborode A.T., Olorunshola M.M., Gbore D., Oladoye M., Adesola R.O., Gbadegoye J.O., Olatoye B.J., Lawal M.A. (2024). The re-emergence and transmission of Monkeypox virus in Nigeria: The role of one health. Front. Public Health.

[9] CDC, “About Mpox,” Mpox. https://www.cdc.gov/mpox/about/index.html.

[10] Musuka G., Moyo E., Tungwarara N., Mhango M., Pierre G., Saramba E., Iradukunda P.G., Dzinamarira T. (2024). A critical review of mpox outbreaks, risk factors, and prevention efforts in Africa: Lessons learned and evolving practices. IJID Reg..

[11] Vaughan A.M., Cenciarelli O., Colombe S., de Sousa L.A., Fischer N., Gossner C.M., Pires J., Scardina G., Aspelund G., Avercenko M. (2022). A large multi-country outbreak of monkeypox across 41 countries in the WHO European Region, 7 March to 23 August 2022. Eurosurveillance.

[12] Mpox (Monkeypox)—Democratic Republic of the Congo. https://www.who.int/emergencies/disease-outbreak-news/item/2023-DON493.

[13] Dukers-Muijrers N.H.T.M., Evers Y., Widdershoven V., Davidovich U., Adam P.C.G., de Coul E.L.M.O., Zantkuijl P., Matser A., Prins M., de Vries H.J.C. (2023). Mpox vaccination willingness, determinants, and communication needs in gay, bisexual, and other men who have sex with men, in the context of limited vaccine availability in the Netherlands (Dutch Mpox-survey). Front. Public Health.

[14] Attitudes towards Receiving Monkeypox Vaccination: A Systematic Review and Meta-Analysis—Abstract—Europe PMC. https://europepmc.org/article/MED/38140243.

[15] Ogunkola I.O., Abiodun O.E., Bale B.I., Elebesunu E.E., Ujam S.B., Umeh I.C., Tom-James M., Musa S.S., Manirambona E., Evardone S.B. (2023). Monkeypox vaccination in the global south: Fighting a war without a weapon. Clin. Epidemiol. Glob. Health.

[16] Zhou Y., Chen Z. (2023). Mpox: A review of laboratory detection techniques. Arch. Virol..

[17] Liu W., Zhang E., Li W., Lv R., Lin Y., Xu Y., Li J., Lai Y., Jiang Y., Lin S. (2024). Advances and challenges of monkeypox detection technology. Biosaf. Health.

[18] Masirika L.M., Udahemuka J.C., Schuele L., Ndishimye P., Otani S., Mbiribindi J.B., Marekani J.M., Mambo L.M., Bubala N.M., Boter M. (2024). Ongoing mpox outbreak in Kamituga, South Kivu province, associated with monkeypox virus of a novel Clade I sub-lineage, Democratic Republic of the Congo, 2024. Eurosurveillance.

[19] WHO Urges Rapid Access to Mpox Diagnostic Tests, Invites Manufacturers to Emergency Review. https://www.who.int/news-room/29-08-2024-who-urges-rapid-access-to-mpox-diagnostic-tests--invites-manufacturers-to-emergency-review.

[20] Alvarez E., Bielska I.A., Hopkins S., Belal A.A., Goldstein D.M., Slick J., Pavalagantharajah S., Wynfield A., Dakey S., Gedeon M.-C. (2023). Limitations of COVID-19 testing and case data for evidence-informed health policy and practice. Health Res. Policy Syst..

[21] World Health Organization [WHO] Ebola Virus Disease. https://www.who.int/news-room/fact-sheets/detail/ebola-virus-disease.

[22] Najimudeen M., Chen H.W.J., Jamaluddin N.A., Myint M.H., Marzo R.R. (2022). Monkeypox in Pregnancy: Susceptibility, Maternal and Fetal Outcomes, and One Health Concept. Int. J. Matern. Child Health AIDS (IJMA).

[23] Schwartz D.A. (2024). High Rates of Miscarriage and Stillbirth among Pregnant Women with Clade I Mpox (Monkeypox) Are Confirmed during 2023–2024 DR Congo Outbreak in South Kivu Province. Viruses.

[24] Rwanda’s New Mobile Lab That Will Boost COVID-19 Testing Capacity. https://www.moh.gov.rw/news-detail/rwandas-new-mobile-lab-that-will-boost-covid-19-testing-capacity.

[25] New Technology Allows for Rapid Diagnosis of Ebola in Democratic Republic of the Congo|WHO|Regional Office for Africa. https://www.afro.who.int/news/new-technology-allows-rapid-diagnosis-ebola-democratic-republic-congo.

[26] Nachega J.B., Mbala-Kingebeni P., Otshudiema J., Mobula L.M., Preiser W., Kallay O., Michaels-Strasser S., Breman J.G., Rimoin A.W., Nsio J. (2020). Responding to the Challenge of the Dual COVID-19 and Ebola Epidemics in the Democratic Republic of Congo—Priorities for Achieving Control. Am. J. Trop. Med. Hyg..

[27] Bosonkie M., Egbende L., Namale A., Fawole O.I., Seck I., Kizito S., Kaba D., Kiwanuka S.N., Diallo I., Bello S. (2023). Improving testing capacity for COVID-19: Experiences and lessons from Senegal, Uganda, Nigeria, and the Democratic Republic of Congo. Front. Public Health.

[28] Sarki A.M., Ezeh A., Stranges S. (2020). Uganda as a Role Model for Pandemic is Containment in Africa. Am. J. Public Health.

[29] Shoemaker T.R., Balinandi S., Tumusiime A., Nyakarahuka L., Lutwama J., Mbidde E., Kofman A., Klena J.D., Ströher U.E., Rollin P. (2018). Impact of enhanced viral haemorrhagic fever surveillance on outbreak detection and response in Uganda. Lancet Infect. Dis..

[30] Republic of Kenya Receives Mobile Labs from the EAC Secretariat. https://www.eac.int/press-releases/147-health/1730-republic-of-kenya-receives-mobile-labs-from-the-eac-secretariat.

[31] Affara M., Lagu H.I., Achol E., Karamagi R., Omari N., Ochido G., Kezakarayagwa E., Kabatesi F., Nkeshimana A., Roba A. (2021). The East African Community (EAC) mobile laboratory networks in Kenya, Burundi, Tanzania, Rwanda, Uganda, and South Sudan—From project implementation to outbreak response against Dengue, Ebola, COVID-19, and epidemic-prone diseases. BMC Med..

[32] Lancet T. (2024). Mpox: The need for a coordinated international response. Lancet.

[33] Alakija A. (2023). Leveraging lessons from the COVID-19 pandemic to strengthen low-income and middle-income country preparedness for future global health threats. Lancet Infect. Dis..

[34] Multi-Country Outbreak of Mpox, External Situation Report#30—25 November 2023. https://www.who.int/publications/m/item/multi-country-outbreak-of-mpox--external-situation-report-30---25-november-2023.

[35] The 4 × 4 Reform: A Path to Quality Health Care in Rwanda. https://www.moh.gov.rw/news-detail/the-4x4-reform-a-path-to-quality-health-care-in-rwanda.

[36] Branda F., Ceccarelli G., Ciccozzi M., Scarpa F. (2024). Strengthening community resilience: Lessons from COVID-19 for mpox prevention. Lancet.

[37] Kozlov M. (2022). Monkeypox outbreaks: 4 key questions researchers have. Nature.

[38] Bertran M., Andrews N., Davison C., Dugbazah B., Boateng J., Lunt R., Hardstaff J., Green M., Blomquist P., Turner C. (2023). Effectiveness of one dose of MVA–BN smallpox vaccine against mpox in England using the case-coverage method: An observational study. Lancet Infect. Dis..

[39] Multi-Country Monkeypox Outbreak Declared a Global Public Health Emergency of International Concern—Africa CDC. https://africacdc.org/news-item/multi-country-monkeypox-outbreak-declared-a-global-public-health-emergency-of-international-concern-2/.

[40] Scaling Up African Vaccine Manufacturing Capacity|Reports|Wellcome. https://wellcome.org/reports/scaling-african-vaccine-manufacturing-capacity.

[41] Lamptey E., Senkyire E.K., Benita D.A., Boakye E.O. (2022). COVID-19 vaccines development in Africa: A review of current situation and existing challenges of vaccine production. Clin. Exp. Vaccine Res..

[42] Li H., Zhang H., Ding K., Wang X.-H., Sun G.-Y., Liu Z.-X., Luo Y. (2022). The evolving epidemiology of monkeypox virus. Cytokine Growth Factor Rev..

[43] Mremi I.R., George J., Rumisha S.F., Sindato C., Kimera S.I., Mboera L.E.G. (2021). Twenty years of integrated disease surveillance and response in Sub-Saharan Africa: Challenges and opportunities for effective management of infectious disease epidemics. One Health Outlook.

[44] Worsley-Tonks K.E.L., Bender J.B., Deem S.L., Ferguson A.W., Fèvre E.M., Martins D.J., Muloi D.M., Murray S., Mutinda M., Ogada D. (2022). Strengthening global health security by improving disease surveillance in remote rural areas of low-income and middle-income countries. Lancet Glob. Health.

[45] Ngum N., Mashingia J., Ndomondo-Sigonda M., Walker S., Salek S. (2022). Evaluation of the Effectiveness and Efficiency of the East African Community Joint Assessment Procedure by Member Countries: The Way Forward. Front. Pharmacol..

[46] Diarra T., Onyeneho N., Okeibunor J., Dialloa B., Yao M.N.K., Djingarey M.H., Fall S., Chamla D., Gueye A.S. (2023). Response of Healthcare Service Providers to the Ebola Virus Disease Epidemic in the Democratic Republic of Congo’s North Kivu and Ituri Provinces. J. Immunol. Sci..

[47] Africa Health Workforce Investment Charter_v6_May 2023.pdf. https://www.afro.who.int/sites/default/files/2023-08/Africa%20Health%20Workforce%20Investment%20Charter_v6_May%202023.pdf.

[48] Haldane V., De Foo C., Abdalla S.M., Jung A.-S., Tan M., Wu S., Chua A., Verma M., Shrestha P., Singh S. (2021). Health systems resilience in managing the COVID-19 pandemic: Lessons from 28 countries. Nat. Med..

[49] Mohammed A., Idris-Dantata H., Okwor T., Tanui P., Paintsil E., Kabwe P.C., Alimi Y., Tajudeen R., Mankoula W., Ilesanmi O.S. (2023). Supporting the Manufacturing of Medical Supplies in Africa: Collaboration Between Africa CDC, Partners, and Member States. Glob. Health Sci. Pract..

[50] ABezruki, Moon S. (2021). Always Fighting the Last War? Post-Ebola Reforms, Blindspots & Gaps in COVID-19. Global Health Centre Working Paper.

[51] Dzinamarira T., Tungwarara N., Chitungo I., Chimene M., Iradukunda P.G., Mashora M., Murewanhema G., Rwibasira G.N., Musuka G. (2022). Unpacking the Implications of SARS-CoV-2 Breakthrough Infections on COVID-19 Vaccination Programs. Vaccines.

[52] Sørensen K., Van den Broucke S., Fullam J., Doyle G., Pelikan J., Slonska Z., Brand H., (HLS-EU) Consortium Health Literacy Project European (2012). Health literacy and public health: A systematic review and integration of definitions and models. BMC Public Health.

[53] Filip R., Puscaselu R.G., Anchidin-Norocel L., Dimian M., Savage W.K. (2022). Global Challenges to Public Health Care Systems during the COVID-19 Pandemic: A Review of Pandemic Measures and Problems. J. Pers. Med..

[54] Nachega J.B., Mohr E.L., Dashraath P., Mbala-Kingebeni P., Anderson J.R., Myer L., Gandhi M., Baud D., Mofenson L.M., Muyembe-Tamfum J.-J. (2024). Mpox in Pregnancy—Risks, Vertical Transmission, Prevention, and Treatment. N. Engl. J. Med..

[55] Muvunyi C.M., Bigirimana N., Tuyishime A., Mukagatare I., Ngabonziza J.C., Ahmed A. (2024). Initiatives and Strategies to Strengthen the National, Regional, and International Global Health Security: A Case Study of Rwanda Biomedical Centre.

[56] Remera E., Rwagasore E., Muvunyi C.M., Ahmed A. (2024). Emergence of the first molecularly confirmed outbreak of Rift Valley fever among humans in Rwanda, calls for institutionalizing the One Health strategy. IJID One Health.

[57] Ahmed A., Daily J.P., Lescano A.G., Golightly L.M., Fasina A. (2020). Challenges and Strategies for Biomedical Researchers Returning to Low- and Middle-Income Countries after Training. Am. J. Trop. Med. Hyg..

